# Thyroxine Reveals Addison’s Disease: A Case Report

**DOI:** 10.7759/cureus.86299

**Published:** 2025-06-18

**Authors:** Vasiliki Rengina Tsinopoulou, Savvas Kolanis, Dimitra Katsarou, Eleni P Kotanidou, Assimina Galli-Tsinopoulou

**Affiliations:** 1 2nd Department of Pediatrics, School of Medicine, Faculty of Health Sciences, Aristotle University of Thessaloniki, AHEPA University General Hospital, Thessaloniki, GRC; 2 Program of Postgraduate Studies Adolescent Medicine and Adolescent Health Care, School of Medicine, Faculty of Health Sciences, Aristotle University of Thessaloniki, AHEPA University General Hospital, Thessaloniki, GRC

**Keywords:** adrenal crisis, autoimmune polyglandular syndrome, hashimoto’s thyroiditis, hypothyroidism, levothyroxine

## Abstract

Autoimmune polyglandular syndrome type 2 (APS-2) is a rare complex autoimmune endocrinological entity and is reported even more rarely in children and adolescents. It consists of primary adrenal insufficiency (Addison’s disease), autoimmune thyroiditis (AIT) and/or type 1 diabetes mellitus (T1DM). Treatment of the AIT component of the disease with levothyroxine as hormone replacement therapy has been reported rarely to cause adrenal crisis. This treatment adverse event is possibly explained due to the increase of basal metabolic rate and subsequently the increased demand and clearance of cortisol after levothyroxine therapy and should always be considered by physicians in order to avoid the development of adrenal crisis. We report the case of a 13-year-old adolescent girl with hypothyroidism due to Hashimoto's thyroiditis (HT) who developed signs of adrenal insufficiency after initial levothyroxine therapy. She was initially admitted with symptoms of hyperthyroidism, including weight loss, anxiety, and oligomenorrhea. A more thorough examination revealed pallor, signs of dehydration, tachycardia, and additionally serum electrolyte imbalance and extremely low cortisol levels in combination with very high adrenocorticotropic hormone (ACTH) levels. The patient received emergency treatment for adrenal insufficiency and recovered well. Due to positive adrenal autoantibodies, APS-2 was confirmed. This case report highlights the necessity of careful adrenal function screening in patients with hypothyroidism and HT before initiating thyroid therapy in order to avoid the rare occurrence of adrenal crisis.

## Introduction

Autoimmune polyglandular syndrome type 2 (APS-2) is an autoimmune endocrinological entity leading to lymphocytic infiltration and subsequent destruction of various organs, often resulting in significant morbidity [1]. Its prevalence is estimated to be 1.5-4.5 cases per 100,000 people, and the syndrome is more commonly manifested in middle-aged women, whereas pediatric cases are exceedingly rare [2]. The mean age at diagnosis is between 35 and 40 years, with a reported female-to-male ratio of approximately 4:1 [1,3]. APS-2 was first described by Schmidt in 1926 [4] and includes primary adrenal insufficiency (PAI; known as Addison’s disease) in all patients combined with autoimmune thyroiditis (AIT; either Graves' disease or Hashimoto’s thyroiditis (HT) and hypothyroidism) and/or type 1 diabetes mellitus (T1DM) [1].

The simultaneous presentation of all three classic manifestations is uncommon, and the interval between the three components might be substantial [5], often delaying diagnosis, which may increase the risk of serious complications. Furthermore, treatment for the AIT component of the disease with levothyroxine as hormone replacement has been reported to cause adrenal crisis [6-9]. In this present study, we report the case of a teenage girl with APS-2 who developed adrenal insufficiency following levothyroxine treatment for hypothyroidism. It is demonstrated that physicians should take caution when administrating levothyroxine for hypothyroidism and HT because in the rare case of APS-2 it could instigate an adrenal crisis.

## Case presentation

A 13-year-old adolescent girl was referred to the Pediatric Endocrinology and Metabolism Unit by a pediatric endocrinologist with clinical and laboratory findings of “hyperthyroidism”. The patient had a prior history of growth hormone deficiency and HT. Following a diagnosis of hypothyroidism, she commenced replacement therapy with levothyroxine, initially at a dose of 25 μg/day, which was subsequently increased to 37 μg/day. She presented with weight loss (3.5 kg in four months), fatigue, anxiety, sleep disturbances, and oligomenorrhea, which, in combination with the laboratory findings, were consistent with hyperthyroidism, and levothyroxine was discontinued. However, despite the discontinuation of levothyroxine, her clinical condition failed to improve, while the patient also presented with vomiting and diarrhea, with deterioration of her clinical state. Repeated laboratory evaluation revealed ongoing hyperthyroidism (thyroid-stimulating hormone (TSH): 0.02 μIU/mL, fT4 (free thyroxine): 2.39 ng/dL, and primary adrenal insufficiency (adrenocorticotropic hormone (ACTH): 1178 pg/mL, cortisol: <1.5 nmol/L) (Table 1).

**Table 1:** Laboratory findings upon admission n.r: normal value, TSH: thyroid-stimulating hormone, fT4: thyroxine, ACTH: adrenocorticotropic hormone, Na: serum sodium, K: serum potassium.

Laboratory tests	Value
TSH (n.r: 0.6-4.8 μIU/mL)	0.02
fT4 (n.r: 0.93-1.7 ng/dL)	2.39
ACTH (n.r: 6-60 pg/mL)	1178
Random cortisol (n.r: 5-25 μg/dL)	<1.5
Na (n.r: 135-145 mmol/L)	112
K (n.r: 3.6-5.2 mmol/L)	3.5
Glucose (n.r: 70-100 mg/dL)	65

Her perinatal history indicated she was the first child of phenotypically healthy parents, delivered by cesarean section at 40 weeks of gestation, with a birth weight of 3 kg. As an adolescent, she had a complete pubertal development and menarche at 12.2 years of age. Due to growth hormone deficiency, she had undergone substitution therapy with recombinant human growth hormone. She was subsequently diagnosed with HT and hypothyroidism, for which she was placed on levothyroxine substitution therapy. Her mother also had HT, while her maternal grandmother had hypothyroidism, for which she received levothyroxine substitution therapy. Additionally, her paternal aunt had been diagnosed with T1DM. Both parents reported normal pubertal development, and her mid-parental target height was 163.5 cm.

On clinical examination, she appeared severely affected, displaying pallor, tachycardia (110 bpm), hypotension (98/38 mmHg), mild hand tremor, dry lips, and coated tongue, findings suggestive of dehydration. There was no apparent skin pigmentation. Mild noticeable palpation of the thyroid gland was also recorded. Her weight was 36.5 kg (5th percentile), height 154 cm (10th-25th percentile), and body mass index 15.39 kg/m^2^ (<5th percentile), indicative of the patient's malnourished state.

Initial laboratory examination revealed severe hyponatremia, mild hypokalemia, hypoglycemia, metabolic acidosis, and dehydration (Table 1). The patient's clinical presentation (pallor, worsening fatigue, anorexia, nausea, vomiting, weight loss) combined with laboratory findings (severe hyponatremia, elevated urea, and creatinine due to dehydration and hemoconcentration, hypocortisolism with high ACTH levels) led to the diagnosis of adrenal insufficiency (Addison’s disease). In addition, clinical and laboratory findings confirmed hyperthyroidism and HT.

The patient was admitted to the Pediatric Department and placed under intensive continuous monitoring of vital signs. She received intravenous fluid resuscitation and intravenous hydrocortisone (initially at a loading dose of 100 mg and then at a maintenance dose of 200 mg hydrocortisone/24 hours divided into four doses). After completion of fluid replacement, intravenous hydrocortisone was transitioned to oral hydrocortisone (10 mg/m^2^/day divided into two doses) and fludrocortisone (100 μg in a single dose). In addition, methimazole was added (initially 0.4 mg per kilogram (kg) of body weight per day, divided into three equal doses and then the dose was adjusted at 0.2 mg per kilogram of body weight per day).

In the context of evaluating the co-occurrence of PAI and hyperthyroidism, a comprehensive diagnostic investigation for APS-2 was performed, involving antibodies against TSH receptor, adrenal glands, gastric parietal cells, glutamic acid decarboxylase IgG, pancreatic islet cells, insulin, zinc transporter ZnT8, and celiac disease serology. Additional assessments included a complete immunological examination, peripheral blood immunophenotype, HLA-DRB1, and HLA-DQB1 antigens (Table 2). TSH receptor antibodies were negative, excluding Graves' disease. Adrenal gland antibodies and 21 hydroxylase antibodies were positive, confirming the diagnosis of autoimmune PAI (Table 2). Celiac disease, T1DM, and pernicious anemia were ruled out.

**Table 2:** Laboratory autoimmunity findings of the female adolescent patient under investigation Ab: antibodies, anti-TPO: peroxidase autoantibodies, anti-TG: thyroglobulin autoantibodies, TSHR Ab: thyroid-stimulating hormone receptor autoantibodies, anti GPC: gastric parietal cell autoantibodies, anti-tTG IgA: transglutaminase immunoglobulin A autoantibodies, anti-GAD: glutamic acid decarboxylase autoantibodies, HLA: human leukocyte antigen.

Laboratory tests	Results
Anti-adrenal cortex Ab	Positive (+)
Anti-21-hydroxylase Ab	Positive (+)
Anti-TPO	Positive (+)
Anti-TG	Positive (+)
TSHR Ab	Negative (-)
Anti-GPC	Negative (-)
Anti-tTG IgA	Negative (-)
Anti-GAD	Negative (-)
Pancreatic islet cells autoantibodies	Negative (-)
Insulin autoantibodies	Negative (-)
Tyrosine phosphatase-like protein	Negative (-)
Zinc transporter 8 autoantibodies	Negative (-)
HLA-DRB1	DRB1*15
HLA-DQB1	DQB1*16, DQB1*05, DQB1*06

During hospitalization, thyroid and abdominal ultrasound examinations were performed and demonstrated normal findings. The patient's clinical condition gradually improved, permitting the discontinuation of intravenous fluid administration, while continuing per os hydrocortisone and fludrocortisone replacement therapy. Methimazole was discontinued very early, as Graves' disease was excluded by the absence of TSH antibodies, HT was confirmed, and thyroid function gradually improved.

## Discussion

APS-2 is a multifactorial disease, caused by both environmental factors and genetic predisposition. Diagnosis requires the presentation of at least two of three different manifestations: PAI or Addison’s disease, AIT, and T1DM [1]. Other associated manifestations may include primary hypogonadism, myasthenia gravis and celiac disease, alopecia, vitiligo, pernicious anemia, idiopathic heart block, Stiff-Man syndrome, Parkinson's disease, IgA deficiency, serositis, dermatitis herpetiformis, idiopathic thrombocytopenia, and hypophysitis [1].

The primary genetic component of APS-2 is the human leukocyte antigen (HLA) haplotype, with HLA-DQ, HLA-DR (HLA-DR3, HLA-DR4), and HLA-DP, being the key alleles involved in APS-2 [10]. Other non-HLA genes such as MICA5.1, CTLA-4, PTPN22, BACH, VTNR, and CD25-IL-2 receptor have also been associated with the syndrome. The HLA-DR3 haplotype is mostly associated with Addison’s disease, with 30% of these patients carrying the HLA DR3/4-DQ2/8 haplotype. In this subset of patients, HLA-DR4 subtype DRB1*0404 confers the highest risk of developing the disease, while the HLA-DR3 haplotype DQA1*0501/DQB1*0201 increases the risk of developing Addison's disease combined with T1DM and celiac disease [10]. Presentation of all three APS-2 manifestations is associated with HLA-DR4 DQA1*0301/DQB1*0302 genotype [10]. Importantly, individuals with genetic predisposition develop antibodies against the enzyme 21-hydroxylase, resulting in gradual loss of cortisol production capability, eventually leading to adrenal insufficiency. Antibody titers against 21-hydroxylase tend to decline as the disease progresses, which increases the diagnostic value of this marker in the early stages. In patients with T1DM, the DRB1*0404 allele and the presence of antibodies against 21-hydroxylase were shown to increase the risk of developing Addison's disease by 100-fold [11]. Our patient had hypothyroidism and also tested positive for autoantibodies against 21-hydroxylase, confirming Addison's disease of autoimmune etiology.

Several reports indicate that initiation of thyroid hormone replacement therapy before glucocorticoid replacement therapy in patients with hypothyroidism and underlying PAI may lead to acute adrenal crisis [6-9,12,13]. A common reason for this complication is delayed diagnosis or misdiagnosis of the PAI [14-16] leading to continuation or even escalation of levothyroxine to treat the patient’s symptoms that resemble hypothyroidism. Even though adrenal crisis is a rare occurrence in patients with adrenal insufficiency, it is a life-threatening condition and physicians should be aware. The presented case adds to the body of literature advocating against the initiation of thyroid hormone replacement therapy prior to adrenal function assessment. The diagnosis of PAI or Addison’s disease is based on morning serum cortisol levels <6.0 mg/dL or <18 mg/dL one hour after stimulation with cosyntropin, along with positive testing for 21-hydroxylase or 17-hydroxylase autoantibodies [1]. Therefore, patients with these autoantibodies should regularly measure serum levels of ACTH and cortisol.

The treatment of APS-2 focuses on managing the underlying autoimmune disorders. AIT, a common manifestation of the syndrome, often presents as HT and leads to hypothyroidism. Hypothyroidism reduces the basal metabolic rate, thus reducing the need for cortisol and additionally, reduces cortisol clearance [17]. Therefore, the mechanism by which thyroid hormone replacement therapy is proposed to precipitate an adrenal crisis is by increasing the metabolic rate while simultaneously promoting cortisol clearance. The increased metabolic rate by thyroxine increases the body’s demand for cortisol, which cannot be met by the adrenal glands, leading to adrenal insufficiency, a life-threatening condition for the patient.

Given the increased risk of overlapping autoimmune conditions, individuals with one endocrine autoimmune disorder are more likely to develop additional autoimmune disorders. Therefore, patients with hypothyroidism due to HT who receive levothyroxine should be screened for adrenal gland function to prevent an adrenal crisis. Moreover, patients with Addison’s disease have a 50% lifetime risk of developing an additional autoimmune disorder [18]; hence, routine screening for associated autoimmune disorders is essential. These patients should also be regularly screened for T1DM and celiac disease. Additionally, first-degree relatives should also be closely monitored.

APS-2 is rarely reported in pediatric populations as shown by the extremely few reports in the literature [18-20], including this clinical case. However, the disease can be life-threatening mostly due to impaired steroidogenesis resulting from the PAI. Therefore, in children with suspected abnormal thyroid function, it is imperative to assess adrenal function prior to initiating thyroxine as replacement therapy to avoid an Addisonian crisis.

## Conclusions

In conclusion, the present case study presented here highlights the need for careful monitoring and adrenal screening of patients with hypothyroidism and HT before initiating levothyroxine therapy, in order to avoid possible adrenal crisis in patients predisposed to autoimmune diseases such as APS-2. Although this clinical presentation is quite rare in the pediatric population, clinicians should be aware of the possibility of the rapidly evolving and life-threatening condition of adrenal crisis.
